# COPPADIS-2015 (COhort of Patients with PArkinson’s DIsease in Spain, 2015), a global –clinical evaluations, serum biomarkers, genetic studies and neuroimaging– prospective, multicenter, non-interventional, long-term study on Parkinson’s disease progression

**DOI:** 10.1186/s12883-016-0548-9

**Published:** 2016-02-25

**Authors:** Diego Santos-García, Pablo Mir, Esther Cubo, Lydia Vela, Mari Cruz Rodríguez-Oroz, Maria José Martí, José Matías Arbelo, Jon Infante, Jaime Kulisevsky, Pablo Martínez-Martín, COPPADIS Study Group

**Affiliations:** Sección de Neurología, Complejo Hospitalario Universitario de Ferrol (CHUF), Hospital Arquitecto Marcide, c/Avenida La Residencia, s/n, 15405 Ferrol, Spain; Unidad de Trastornos del Movimiento, Servicio de Neurología y Neurofisiología Clínica, Instituto de Biomedicina de Sevilla, Hospital Universitario Virgen del Rocío, CSIC y Universidad de Sevilla, Sevilla, Spain; Centro de Investigación Biomédica en Red sobre Enfermedades Neurodegenerativas (CIBERNED), Sevilla, Spain; Servicio de Neurología, Hospital Universitario de Burgos, Burgos, Spain; Unidad de Neurología, Fundación Hospital de Alcorcón, Madrid, Spain; Instituto de Investigación Biodonostia, Hospital Universitario Donostia, San Sebastián, Spain; Unidad de Parkinson y Trastornos del Movimiento, Servicio de Neurología, Instituto Clínico de Neurociencias, Hospital Clínic, Barcelona, Spain; Unidad de Trastornos del Movimiento y enfermedad de Parkinson, Servicio de Neurología, Hospital Universitario Insular de Gran Canaria, Las Palmas de Gran Canaria, Spain; Unidad de Trastornos del Movimiento, Servicio de Neurología, Hospital Universitario Marqués de Valdecilla, Santander, Spain; Unidad de Trastornos del Movimiento, Servicio de Neurología, Hospital de Sant Pau, Barcelona, Spain; Centro Nacional de Epidemiología, Instituto de Salud Carlos III, Madrid, Spain

**Keywords:** Biomarkers, Caregiver, Genetic studies, Magnetic resonance imaging, Non-motor symptoms, Parkinson’s disease, Progression, Quality of life

## Abstract

**Background:**

Parkinson’s disease (PD) is a progressive neurodegenerative disorder causing motor and non-motor symptoms that can affect independence, social adjustment and the quality of life (QoL) of both patients and caregivers. Studies designed to find diagnostic and/or progression biomarkers of PD are needed. We describe here the study protocol of COPPADIS-2015 (COhort of Patients with PArkinson’s DIsease in Spain, 2015), an integral PD project based on four aspects/concepts: 1) PD as a global disease (motor and non-motor symptoms); 2) QoL and caregiver issues; 3) Biomarkers; 4) Disease progression.

**Methods/design:**

Observational, descriptive, non-interventional, 5-year follow-up, national (Spain), multicenter (45 centers from 15 autonomous communities), evaluation study. Specific goals: (1) detailed study (clinical evaluations, serum biomarkers, genetic studies and neuroimaging) of a population of PD patients from different areas of Spain, (2) comparison with a control group and (3) follow-up for 5 years. COPPADIS-2015 has been specifically designed to assess 17 proposed objectives. Study population: approximately 800 non-dementia PD patients, 600 principal caregivers and 400 control subjects. Study evaluations: (1) baseline includes motor assessment (e.g., Unified Parkinson’s Disease Rating Scale part III), non-motor symptoms (e.g., Non-Motor Symptoms Scale), cognition (e.g., Parkinson’s Disease Cognitive Rating Scale), mood and neuropsychiatric symptoms (e.g., Neuropsychiatric Inventory), disability, QoL (e.g., 39-item Parkinson’s disease Quality of Life Questionnaire Summary-Index) and caregiver status (e.g., Zarit Caregiver Burden Inventory); (2) follow-up includes annual (patients) or biannual (caregivers and controls) evaluations. Serum biomarkers (S-100b protein, TNF-α, IL-1, IL-2, IL-6, vitamin B12, methylmalonic acid, homocysteine, uric acid, C-reactive protein, ferritin, iron) and brain MRI (volumetry, tractography and MTAi [Medial Temporal Atrophy Index]), at baseline and at the end of follow-up, and genetic studies (DNA and RNA) at baseline will be performed in a subgroup of subjects (300 PD patients and 100 control subjects). Study periods: (1) recruitment period, from November, 2015 to February, 2017 (basal assessment); (2) follow-up period, 5 years; (3) closing date of clinical follow-up, May, 2022. Funding: Public/Private.

**Discussion:**

COPPADIS-2015 is a challenging initiative. This project will provide important information on the natural history of PD and the value of various biomarkers.

## Background

Parkinson’s disease (PD), the second most common neurodegenerative disease after Alzheimer’s disease, is a progressive neurodegenerative disorder causing motor and non-motor symptoms that result in disability, loss of patient autonomy and caregiver burden [1]. Understanding of PD has changed over recent years, with the disease currently considered to be a neurodegenerative disease involving a diversity of pathways and neurotransmitters that may explain, in part, the large range of symptoms that patients may have [2]. PD is not only a motor disease, as it also involves various non-motor symptoms that are important for different reasons. Non-motor symptoms are frequent and disabling, therefore, early identification and proper management of these symptoms is important [3]. Some non-motor symptoms (e.g., olfactory disorders, constipation or sleep disturbances) may precede motor symptoms, and could be useful as prodromal/preclinical markers of PD [4]. Others, such as dementia and psychosis, are more frequently develop during the late stages of the disease and sometimes difficult to manage. We need to know, in detail, the progression of non-motor symptoms and their relationship with motor changes over time. Reliable and well-validated biomarkers for PD to identify individuals “at risk” before motor symptoms develop, to accurately diagnose individuals at the threshold of clinical PD, and to monitor PD progression (motor and non-motor symptoms) throughout the course of the disease would dramatically accelerate research into both the cause and treatment of PD [5].

Although the identification of a marker for diagnosis and for disease progression (preferably one that is non-invasive, affordable and accessible) is of utmost importance, concepts like quality of life (QoL) are also very important in chronic diseases, such as PD, for which a cure does not exist [6]. Improving patient QoL and identifying factors that lead to caregiver burden are very important aspects of the management of PD. In particular, the role of the principal caregiver in PD is very important because caregiver burden generates poor care and, in the long term, leads to patient institutionalization [7]. Specifically, identifying the changes experienced by PD patients and their caregivers in their QoL and degree of burden, respectively, over time, as well as factors that may predict these changes, in order to carry out a proper intervention, should be a priority. Well-designed, longitudinal prospective studies are key. Access to a population with a high proportion of patients who have been assessed comprehensively and rigorously, without screening bias, is highly valuable for both cross-sectional analysis and prospective follow-up. This is especially relevant for studying populations affected by a neurodegenerative disease, given that these patients are expected to develop different complications that we could identify and analyze.

Studies designed to identify PD diagnostic and/or progression biomarkers and to elucidate the natural progression of the disease are needed. We describe here the COPPADIS-2015 (COhort of Patients with PArkinson’s DIsease in Spain, 2015) study protocol, an integral PD project based on four aspects/concepts: 1) PD as a global disease (motor and non-motor symptoms); 2) QoL and caregiver issues; 3) Biomarkers; 4) Disease progression.

## Methods/design

### Type of study

COPPADIS-2015 is a national, multicenter, epidemiological, descriptive, observational, non-interventional, longitudinal-prospective, 5-year follow-up study. COPPADIS-2015 has been classified by the AEMPS (*Agencia Española del Medicamento y Productos Sanitarios*) as a Post-authorization Prospective Follow-Up study.

The study will be conducted at different hospital sites in Spain. The essential requirement will be that the Principal Investigator participating at each site has experience and skills in the diagnosis and standard management of patients with PD in their daily clinical practice. As such, the participating site could be any establishment from a highly-specialized Movement Disorders Unit of a tertiary hospital to a general neurologist from a regional hospital with the aforementioned skills. More than one hundred researchers from 48 centers in Spain, from 15 autonomous communities, will participate in this project.

### Specific goals

The aim COPPADIS-2015 is to: (1) study in detail (clinical evaluations, serum biomarkers, genetic studies and neuroimaging) a population of patients with PD representative of different areas of Spain; (2) compare it with a control group and (3) follow-up for 5 years. COPPADIS-2015 has been specifically designed to assess 17 proposed objectives (Table 1).

**Table 1:** Specific objectives of the COPPADIS-2015 project

1)	To study what variables (non-motor symptoms, cognition, neuropsychiatric symptoms, falls, disability, etc.) impact negatively on both overall and health-related QoL of patients with PD.
2)	To study what variables contribute to a worse QoL, mood and the burden of the primary caregiver for the patient with PD, as well as if the latter have repercussions on the mood and QoL of the patient him/herself.
3)	To study the frequency of impulse control disorders and their types in patients with PD, as well as what variables are associated with them and to compare it against a control group.
4)	To study the frequency of different non-motor symptoms in patients with PD and to compare it against a control group.
5)	To study the frequency of pain (and its types) [90] in patients with PD, its relationship to the disease and different variables and to compare it against a control group.
6)	To study the frequency of different types of mood disorders (major depression, minor depression, subclinical depression) [91, 92] in patients with PD, its relationship with other disease variables and to compare it against a control group.
7)	To study the different types of parkinsonian phenotypes [93, 94] and their relationship with other variables (clinical, molecular, genetic and neuroimaging).
8)	To study the relationship between different variables (clinical, molecular, genetic and neuroimaging) and motor laterality asymmetry [95].
9)	To study the relationship between the serum levels of S-100b protein, TNF-ɑ, IL-1, IL-2, IL-6, vitamin B12, methylmalonic acid, homocysteine, uric acid, C-reactive protein, ferritin and iron and other disease variables in patients with PD and to compare it against a control group.
10)	To perform genetic studies on DNA and RNA extracted from the lymphocytes of peripheral blood samples.
11)	To study the possible value of a recently proposed imaging marker (MTAi) [30] to detect cognitive alterations in patients with PD and to compare it against a control group.
12)	To perform volumetric imaging and tractography studies to find correlations, under plausible hypotheses, between the neuroimaging parameters and the clinical or neuropsychiatric variables and/or other variables covered in this study [59–61].
13)	To study the incidence of acute hospitalization throughout the 12 months following the baseline assessment (for each patient), their causes, and predictive factors in patients with PD and to compare it against a control group.
14)	To study what percentage of patients with PD develop motor complications (in the subgroup of those who do not present them at the baseline assessment) throughout the 48 months following the baseline assessment (for each patient) and to identify predictive factors.
15)	To study what percentage of patients with PD develop significant cognitive impairment and/or dementia over the course of the 24, 48 and 60 months following the baseline assessment (for each patient), to compare it against a control group and to identify predictive factors (clinical, molecular, genetic, imaging).
16)	To study the morbidity and mortality of patients with PD throughout the 24 and 60 months following the baseline assessment (for each patient), to compare it against a control group and to identify predictive factors (clinical, molecular, genetic, imaging).
17)	At the end of follow-up, to compare the course of the different clinical (motor and no-motor features) and paraclinical variables (molecular and imaging markers).

*IL* interleukin, *PD* parkinson’s disease, *MTAi*, medial temporal atrophy index, *QoL* quality of life, *TNF* tumor necrosis factor

### Study population

Non-dementia patients with idiopathic PD, caregivers (patient’s primary caregiver, if applicable) and controls will be assessed. We plan to include:

1) Approximately 800 patients with PD. Patients will be included if they have idiopathic PD according to the United Kingdom Parkinson’s Disease Society Brain Bank criteria [8], have no dementia criteria (Mini Mental State Examination [MMSE] ≥ 26) [9], are aged between 30 and 75 years, are participating voluntarily and have provided written informed consent. Patients will be excluded from the study if they: (1) are not capable of completing the questionnaires adequately; (2) have other disabling concomitant neurological disease (stroke, severe head trauma, neurodegenerative disease, etc.); (3) have other severe and disabling concomitant non-neurological disease (oncological, autoimmune, etc.); (4) have known chronic anemia and/or hyperuricemia; (5) are receiving active treatment with continuous infusion of levodopa and/or apomorphine and/or with deep brain stimulation; (6) they are participating in a clinical trial and/or other type of study that does not permit concomitant participation in another or (7) if long-term follow-up is not expected to be possible.

2) Approximately 600 caregivers (patient’s primary caregiver). A person who, without being a professional and/or receiving money in exchange for services, lives with the patient and is responsible for his/her care will be included as a primary caregiver [10]. He or she must voluntarily agree to participate and provide written informed consent.

3) Approximately 400 control subjects matched by age, sex and educational level. The control subject could be a family member (not the patient’s caregiver) or friend of the patient who would like to participate voluntarily. The same inclusion criteria (except PD diagnosis) and exclusion criteria as those for the patients will be applied.

### Study design

The study will be carried out in two phases:

1. Cross-sectional assessment (recruitment of patients with a baseline assessment over a period of 16 months, from November 2015 to February 2017). We have also considered the possibility, if necessary, of extending the recruitment period up to a maximum of 24 months in order to achieve the proposed target sample size.
2. Prospective follow-up of the defined cohort of PD patients (COPPADIS) over 5 years.

### Study assessments

The Principal Investigator will make the decision on the patient’s inclusion. Extensive information on sociodemographic aspects, factors related to PD, comorbidity and treatment will be collected. Patient baseline evaluations will include motor assessment (*Hoenh & Yahr* [11], *Unified Parkinson*’*s Disease Rating Scale* [*UPDRS*] *part III and part IV* [12], *Freezing of Gait Questionnaire* [*FOGQ*] [13]), non-motor symptoms (*Non*-*Motor Symptoms Scale* [*NMSS*] [14], *Parkinson*’*s Disease Sleep Scale* [*PDSS*] [15], *Visual Analog Scale*-*Pain* [*VAS*-*Pain*] [16], *Visual Analog Fatigue Scale* [*VAFS*] [17]), cognition (*MMSE* [18], *Parkinson*’*s Disease Cognitive Rating Scale* [*PD*-*CRS*] [19], completing a simple 16-piece puzzle [20]), mood and neuropsychiatric symptoms (*Beck Depression Inventory*-*II* [*BDI*-*II*] [21], *Neuropsychiatric Inventory* [*NPI*] [22], *Questionnaire for Impulsive*-*Compulsive Disorders in Parkinson*’*s Disease*-*Rating Scale* [*QUIP*-*RS*] [23]), disability (*Schwab & England Activities of Daily Living Scale* [*ADLS*] [24]) and QoL (*39*-*item Parkinson*’*s disease Quality of Life Questionnaire Summary Index* [*PDQ*-*39SI*] [25], *PQ*-*10* [26], European Health Interview Survey-Quality of Life *8 item index* [*EUROHIS*-*QOL 8 item*-*index*] [27]). Caregiver baseline evaluation includes the degree of burden (Z*arit Caregiver Burden Inventory* [*ZCBI*] [28], *Caregiver Strain Index* [*CSI*] [29]), mood (*BDI*-*II*) and QoL (*PQ*-*10*, *EUROHIS*-*QOL 8 item*-*index*). The same evaluation as for the patients, except for the motor assessment, will be performed in control subjects at baseline.

In a subgroup of 400 consecutive non-selected subjects (300 PD patients and 100 controls) willing to participate voluntarily and without any contraindications (e.g., magnetic resonance imaging [MRI]), complementary tests will be performed as follows:

1. Blood sample collection for the determination of different molecular biomarkers: S-100b protein, tumor necrosis factor (TNF)-ɑ, interleukin (IL)-1, IL-2, IL-6, vitamin B12, methylmalonic acid, homocysteine, uric acid, C-reactive protein, ferritin and iron. The analysis will be conducted at a common laboratory: REFERENCE LABORATORY (www.reference-laboratory.es). The extraction of the sample will be carried out no longer than 3 months after the first clinical assessment.
2. Blood sample collection for genetic studies on lymphocyte DNA and RNA with 4 fundamental objectives: (1) expression profile studies; (2) exome sequencing (*SNCA*, *LRRK2*, *FBXO7*, *PINKI*, *PRKN*, *DJ1*, *HTRA2*, *UCHLI*, *ATPI3A2*, *VPS35*, *PLA2G6*, *GIGYF2*, *EIF4G1*, *GBA*); (3) analysis of genes considered relevant according to the state of the art; (4) candidate gene association studies in order to determine if the pathological variations are present more frequently in subjects with PD (with respect to the control group).
3. Neuroimaging study: T1 3D MRI of the head using standardized protocols that include frontal and/or coronal slices in T1 sequence at 1.5 T or above without intravenous contrast. Volumetric (spoiled gradient recalled sequence; TR 8,5 ms, TE 4 ms, flip angle 8°, FOV 240 × 240 mm^2^, thickness 1 mm, matrix 288 × 288, voxel size 0.84 mm^3^) and tractography (echo-planar imaging; TR 9.500 ms, TE 73 ms, FOV 224, thickness 2 mm, matrix 128 × 128, b-factors of zero and 700 s/mm^2^) studies will be performed. Also, the Medial Temporal Atrophy Index (MTAi) [30] will be calculated. The MRI study will be performed no longer than 6 months after the first clinical assessment.

**Fig. 1:**
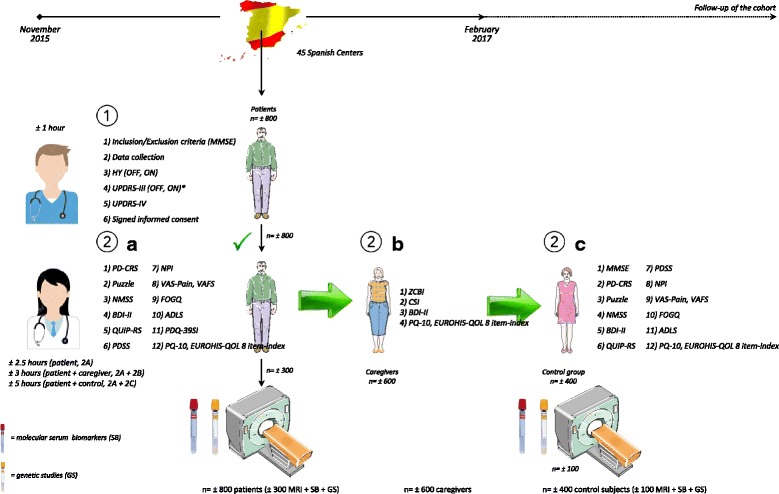
Recruitment period, from November 2015 to February 2017 (baseline assessment of each participating subject). 1, patient inclusion process and motor assessment by the Principal Investigator (neurologist who is an expert in movement disorders and Parkinson’s disease); 2**a**, non-motor assessment by the Principal Investigator, specialized nurse, psychologist or fellow with adequate training; 2**b**, caregiver assessment; 2**c**, control assessment. *Only patients with motor fluctuations (UPDRS-IV) will be assessed during the OFF-medication (first thing in the morning without taking medication in the 12 previous hours) and during the ON-medication state; the rest will only be assessed during the OFF-medication state. Blood samples from the baseline assessment will be stored for the purpose of being able to test other future markers not currently included in the project

Figure 1 shows baseline study assessments. Table 2 shows data regarding centers, number of patients that they have estimated to recruit and distribution of patients selected for the complementary studies according to the different centers.

**Table 2:** Centers, number of patients that they have estimated to recruit and distribution of patients selected for the complementary studies according to the different centers

Principal Investigator; PI’s participating site		City	Patients (n)	Controls (n)	Complementary studies (PDP/CS)
1.	Diego Santos García; Neurology Section, Hospital Arquitecto Marcide, Complejo Hospitalario Universitario de Ferrol (CHUF).	Ferrol (A Coruña)	60	10	45/10
2.	(1) Oriol de Fábregues-Boixar Nebot and (2) Jorge Hernández Vara; Movement Disorders Unit, Neurology Service, Hospital Universitario Vall d’Hebron.	Barcelona	60 (40/20)	20 (10/10)	40/20
3.	Carmen Borrue Fernández; Movement Disorders Unit, Neurology Service, Hospital Infanta Sofía.	Madrid	50	10	NO
4.	Pablo Mir Rivera; Movement Disorders Unit, Neurology and Clinical Neurophysiology Service, Instituto de Biomedicina de Sevilla, Hospital Universitario Virgen del Rocío, CSIC and Universidad de Sevilla.	Seville	40	10	40/0
5.	Maria José Martí Domenech; Parkinson’s and Movement Disorders Unit, Neurology Service, Instituto Clínico de Neurociencias, Hospital Clínic.	Barcelona	40	10	NO
6.	Miquel Aguilar Barberá; Movement Disorders Unit, Hospital Universitario Mutua de Terrassa.	Barcelona	40	10	30/10
7.	Beatriz Tijero Merino; Functional Neurology and Parkinson’s Disease Unit, Hospital de Cruces.	Bilbao	35-40	10	NO
8.	José Chacón Peña; Neurology Unit, Hospital Infanta Luisa.	Seville	30-35	10	NO
9.	(1) Manuel Seijo Martínez and (2) Iria Cabo López; Neurology Section, Hospital de Pontevedra.	Pontevedra	30 (15/15)	20 (10/10)	NO
10.	Víctor Puente Périz; Movement Disorders Unit, Neurology Service, Hospital del Mar.	Barcelona	25-30	10	20/10
11.	Inés Legarda Ramiréz; Neurology Service, Hospital Universitario Son Espases.	Palma de Mallorca	25	10	NO
12.	Francisco Carrillo Padilla; Neurology Service, Hospital Universitario de Canarias, San Cristóbal de la Laguna.	Santa Cruz de Tenerife	25	10	20/0
13.	Lydia López Manzanares; Movement Disorders Unit, Neurology Service, Hospital La Princesa.	Madrid	25	10	NO
14.	Caridad Valero Merino; Neurology Unit, Hospital Arnau de Vilanova.	Valencia	20-25	10	NO
15.	Jaime Kulisevsky Bojarski; Movement Disorders Unit, Neurology Service, Hospital de Sant Pau.	Barcelona	20	10	NO
16.	José Manuel García Moreno; Movement Disorders Unit, Hospital Universitario Virgen Macarena.	Seville	20	10	20/10
17.	Benito Galeano Bilbao; Neurology Section, Hospital Universitario de Ceuta.	Ceuta	20-25	10	20/0
18.	Nuria Caballol Pons; Movement Disorders Unit, Consorci Sanitari Integral, Hospital Moisés Broggi.	Sant Joan Despí	20	10	NO
19.	Mari Cruz Rodríguez Oroz; Hospital Universitario Donostia, Instituto de Investigación Biodonostia.	San Sebastián	20	10	20/10
20.	María Iciar Gastón Zubimendi; Movement Disorders Unit, Neurology Service, Complejo Hospitalario de Navarra.	Pamplona	20	10	NO
21.	Pilar Sánchez Alonso; Neurology Service, Hospital Puerta de Hierro.	Madrid	15-25	10	NO
22.	Esther Cubo Delgado; Neurology Service, Complejo Asistencial Universitario de Burgos.	Burgos	15	10	NO
23.	Lydia Vela Desojo; Neurology Unit, Fundación Hospital de Alcorcón.	Alcorcón (Madrid)	15	10	NO
24.	Maria José Catalán Alonso; Movement Disorders Unit, Neurology Service, Hospital Clínico San Carlos.	Madrid	15	10	NO
25.	Luis Manuel López Díaz; Neurology Section, Hospital de Burela.	Burela (Lugo)	15	10	NO
26.	Maria Gema Alonso Losada; Neurology Service, Hospital Meixoeiro, Complejo Hospitalario Universitario de Vigo (CHUVI).	Vigo (Pontevedra)	15	10	NO
27.	Nuria López Ariztegui; Movement Disorders Unit, Complejo Hospitalario de Toledo.	Toledo	15	10	NO
28.	Mónica Kurtis Urra; Movement Disorders Unit, Neurology Service, Hospital Ruber Internacional.	Madrid	15	10	NO
29.	Jon Infante Ceberio; Movement Disorders Unit, Neurology Service, Hospital Universitario Marqués de Valdecilla.	Santander	15	10	15/10
30.	Sonia Escalante Arroyo; Neurology Service, Hospital de Tortosa Verge de la Cinta (HTVC).	Tortosa (Tarragona)	15	10	15/10
31.	Juan Carlos Martínez Castrillo; Movement Disorders Unit, Neurology Service, Hospital Ramón y Cajal.	Madrid	15	10	NO
32.	José Matías Arbelo González; Movement Disorders and Parkinson’s Disease Unit, Neurology Service, Hospital Universitario Insular de Gran Canaria.	Las Palmas de Gran Canaria	15	10	NO
33.	René Ribacoba Montero; Movement Disorders Unit, Neurology Service, Hospital Central de Asturias.	Oviedo	15	10	15/10
34.	Jessica González Ardura; Neurology Service, Hospital Universitario Lucus Augusti (HULA).	Lugo	15	10	NO
35.	Javier López del Val; Movement Disorders Unit, Neurology Service, Hospital Clínico Universitario Lozano Blesa.	Zaragoza	15	10	NO
36.	María Asunción Ávila Rivera; Movement Disorders Unit, Consorci Sanitari Integral, Hospital General de L’Hospitalet.	L’Hospitalet de Llobregat	15	10	NO
37.	Hortensia Alonso Navarro; Neurology Section, Hospital Universitario del Sureste, Madrid.	Madrid	15	10	NO
38.	Berta Solano Vila; Neurology Service, Hospital Josep Trueta and Parc Martí i Juliá, Girona.	Girona	15	10	NO
39.	Juan García Caldentey; Neurology Unit, Hospital Quirón Palmaplanas.	Palma de Mallorca	15	10	NO
40.	Ana Rojo Sebastián; Parkinson’s and Abnormal Movement Unit, Neurology Service, Hospital Universitario Príncipe de Asturias.	Alcalá de Henares (Madrid)	15	10	NO
41.	Silvia Martí Martínez; Neurology Service, Hospital General de Alicante.	Alicante	15	10	NO
42.	José Andrés Domínguez Morán; Neurology Unit, Hospital de la Rivera.	Alcira (Valencia)	15	10	NO
43.	Irene Martínez Torres; Movement Disorders Unit, Neurology Service, Hospital La Fe.	Valencia	15	10	NO
44.	María Álvarez Sauco; Neurology Service, Hospital General Universitario de Elche.	Elche (Alicante)	15	10	NO
45.	Cristina Prieto Jurczynska; Movement Disorders Unit, Hospital Infanta Elena-Hospital Rey Juan Carlos-Hospital Collado Villalba, Madrid.	Madrid	15	10	NO
			1.000	470	300/100

*PDP* parkinson’s disease patient, *CS* control subject

**Fig. 2:**
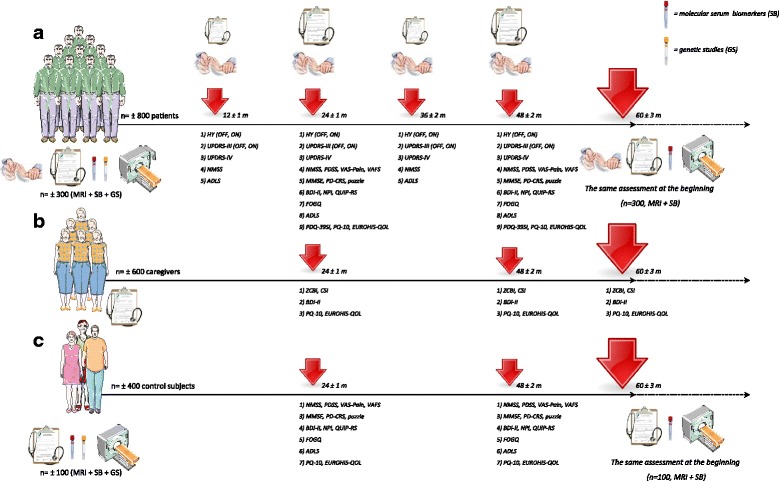
Follow-up of the cohort of patients (**a**), caregivers (**b**) and controls (**c**). At the end of follow-up, the patients (n = 300) and controls (n = 100) who underwent complementary tests at baseline will undergo repeat testing (molecular markers and imaging study). ADLS, Schwab & England Activities of Daily Living Scale; BDI, Beck Depression Inventory; CSI, Caregiver Strain Index; FOGQ, Freezing of Gait Questionnaire; HY, Hoenh & Yahr; NMSS, Non-Motor Symptoms Scale; NPI, Neuropsychiatric Inventory; PD-CRS, Parkinson’s Disease Cognitive Rating Scale; PDQ-39SI, 39-item Parkinson’s disease Quality of Life Questionnaire Summary Index; QUIP-RS, Questionnaire for Impulsive-Compulsive Disorders in Parkinson’s Disease-Rating Scale; PDSS, Parkinson’s Disease Sleep Scale; UPDRS, Unified Parkinson’s Disease Rating Scale; VAFS, Visual Analog Fatigue Scale; VAS-Pain, Visual Analog Scale-Pain; ZCBI, Zarit Caregiver Burden Inventory

During the 5 years of follow-up, annual evaluations (PD patients) or evaluations at 24, 48 and 60 months (caregivers and controls) will be performed. Figure 2 shows assessments during the follow-up phase in each group. The subjects who undergo complementary tests at baseline will repeat the head MRI study (preferably at the same site and with the same machine) and determination of molecular markers (S-100b, TNF-ɑ, IL-1, IL-2, IL-6, vitamin B12, methylmalonic acid, homocysteine, uric acid, C-reactive protein, ferritin and iron; same laboratory, REFERENCE LABORATORY) at 60 months. Table 3 shows the working plan summary.

**Table 3:** Working Plan Summary

Assessments	Baseline	12 m	24 m	36 m	48 m	60 m
Inclusion/exclusion criteria	P; C					
Hoehn & Yahr	P	P	P	P	P	P
UPDRS-III and UPDRS-IV	P	P	P	P	P	P
MMSE	P; C		P; C		P; C	P; C
PD-CRS	P; C		P; C		P; C	P; C
Puzzle test	P; C		P; C		P; C	P; C
NMSS	P; C	P	P; C	P	P; C	P; C
BDI-II	P; pC; C		P; pC; C		P; pC; C	P; pC; C
QUIP-RS	P; C		P; C		P; C	P; C
PDSS	P; C		P; C		P; C	P; C
NPI	P; C		P; C		P; C	P; C
VAS-Pain and VAFS	P; C		P; C		P; C	P; C
FOGQ	P; C		P; C		P; C	P; C
ADLS	P; C	P	P; C	P	P; C	P; C
PDQ-39SI	P; C		P; C		P; C	P; C
PQ-10	P; pC; C		P; pC; C		P; pC; C	P; pC; C
EUROHIS-QOL 8 item index	P; pC; C		P; pC; C		P; pC; C	P; pC; C
ZCBI	pC		pC		pC	pC
CSI	pC		pC		pC	pC
Samples-serum markers	P; C					P; C
Samples-genetic studies	P; C					
Head MRI	P; C					P; C

*ADLS* Schwab & England activities of daily living scale, *BDI* beck depression inventory, *C* control, *CSI* caregiver strain index, *EUROHIS*-*QOL* European health interview survey-quality of life, *FOGQ* freezing of gait questionnaire, *m* month, *MMSE* mini mental state examination, *MRI* magnetic resonance imaging, *NMSS* non-motor symptoms scale, *NPI* neuropsychiatric inventory, *P* patient, *pC* primary caregiver, *PD*-*CRS* parkinson’s disease cognitive rating scale, *PDQ*-*39SI* 39-item parkinson’s disease quality of life questionnaire summary index, *PDSS* parkinson’s disease sleep scale, *QUIP*-*RS* questionnaire for impulsive-compulsive disorders in parkinson’s disease-rating scale, *UPDRS* unified parkinson’s disease rating scale, *VAFS* visual analog fatigue scale, *VAS*-*Pain* visual analog scale-pain, *ZCBI* zarit caregiver burden inventory

### Data collection and statistical analysis

Data will be collected using an electronic Case Report Form (e-CRF) and will be transferred to a statistical package for subsequent analysis. The company responsible for study monitoring is Alphabioresearch (www.alphabioresearch.com).

We calculated the sample size, taking into account a maximum estimated loss to follow-up of 10 % of patients per year, and that between 15 % and 20 % of the sites involved in a multicenter study are estimated to leave the study for different reasons [31]. A minimum of 280 patients at the end of follow-up would be sufficient to include up to 27 predictive variables in the multiple regression analyses with a power of 0.8 and a confidence interval of 95 % [32]. The pertinent analysis (descriptive studies, univariate studies, binary logistic regression, multiple linear regression, etc.) will be performed based on the type of objective. In addition, given de complexity of potential analysis including a diversity of variables from different origin and measurement properties, advanced statistical methodology (data mining, artificial intelligence techniques, etc.) will be applied as needed. One of the authors (PMM) will coordinate these aspects.

### Ethical considerations

The project will be conducted in accordance with the standards for Good Clinical Practice, the fundamental ethical principles established in the Declaration of Helsinki and the Oviedo Convention, as well as the requirements established in Spanish legislation in the research field. Approval of the Ethics Committee at each center was obtained.

### Study timetable

1. Pre-start-up procedures: until October 2015.
2. First assessment (cross-sectional assessment): November 2015 to February 2017.
3. Database review: March–June 2017.
4. Statistical analysis: second half of 2017.
5. Reporting and publication of papers (objectives 1 to 12 of the cross-sectional assessment and other subanalyses): years 2017 to 2019.
6. Objectives of the prospective follow-up of the cohort: from 2018 onwards.

### Future possibilities of the project

1. To perform other evaluations and/or complementary studies during follow-up; for example, electromyographic studies to determine the frequency of polyneuropathy in PD patients compared with controls and also to identify related risk factors (clinical, biomarkers, etc.); other studies (optical coherence tomography, electroencephalogram, salivary secretion and/or cerebrospinal fluid analysis, skin biopsy, etc.).
2. To continue follow-up of the subjects (patients, caregivers and controls) over time (10, 15, 20 years, etc.).
3. To create a COPPADIS Brain Bank. We have designed a working group with the participation of members of different biobanks in Spain with the aim of create a Brain Donation Program for COPPADIS PD patients.

## Discussion

The described project, COPPADIS-2015 (COhort of Patients with PArkinson’s DIsease in Spain, 2015), is an ambitious initiative that will provide important information regarding the natural history of PD and the value of various biomarkers. The large sample size, high level of participation, with more than 40 Spanish centers involved, exhaustive clinic evaluations, including motor and non-motor features (more than 500 variables collected from the baseline assessment), biomarkers, study design and interesting future possibilities are strong points of this project. COPPADIS-2015 will enable us to identify different complications that develop over time in a very large population of patients with PD. Therefore, we expect to be able to identify the incidence of different problems, their impact on QoL and predictive factors that will allow us to identify these problems early in order to act.

This project is based on four aspects/concepts: 1) PD as a global disease (motor and non-motor symptoms); 2) QoL and caregiver issues; 3) Biomarkers; 4) Disease progression. For years, PD management was focused on motor symptoms. However, more recently, non-motor features have been gaining in importance. Non-motor symptoms are important for different reasons [33]: they are frequent and disabling; some non-motor symptoms such as hyposmia, constipation, depression or REM sleep behavior disorders can precede motor symptoms and, in the future, such symptoms could be used to establish an earlier diagnosis of premotor PD; non-motor symptoms can sometimes be difficult to manage (orthostatic hypotension, behavioral disorders, etc.); some non-motor symptoms (dementia, psychosis, etc.) increase the risk of institutionalization and generate a high economic cost; and they are not always sufficiently recognized by the neurologist. Additionally, various studies have demonstrated that non-motor symptoms impact negatively on patient QoL, and that by improving these symptoms, we help improve QoL [34, 35]. Early identification and proper management should currently be a priority in daily clinical practice [4], but for many reasons, this is not always the case. In COPPADIS-2015, we will exhaustively evaluate different non-motor features (*NMSS*, *PDSS*, *VAS*-*Pain*, *VAFS*, *MMSE*, *PD*-*CRS*, *BDI*-*II*, *NPI*, *QUIP*-*RS*) in a large population of PD patients. We will also compare these features with a control group and analyze their evolution with disease progression. Finally, we will try to identify related (cross-sectional assessment) and predictive (prospective follow-up) factors for the development of different non-motor symptoms. For example, factors related to mild cognitive impairment at baseline and risk factors that could predict the chance of mild cognitive impairment progressing to dementia will be identified [36]. We will also compare non-motor symptoms (and other features of the disease) and disease progression in patients with different phenotypes and motor laterality asymmetry. Moreover, we will try to determine if non-motor symptoms (NMSS) predict acute hospitalization in PD patients [37].

At present we have no cure for PD. We use different therapies to improve patient symptoms, health status, degree of autonomy and QoL. The key is to understand what factors affect QoL (depression, pain, motor complications, etc.), given that interventions will be based on these factors. Moreover, the role of the patient’s primary caregiver is also very important because PD is a neurodegenerative disorder and the patient will become increasingly dependent. We will need to determine which factors cause caregivers’ stress and increase their burden in order to carry out an early intervention, since caregiver burden generates poor care and, in the long term, often leads to the patient’s institutionalization [7]. In this regard, it has been observed that patient QoL is correlated with the caregiver’s status, and interventions targeted at improving caregiver burden to also improve the patient’s QoL have been suggested [38]. Although there are several studies focused on factors related to caregiver burden [39–41], there is no information about caregiver strain changes during disease progression. In COPPADIS-2015 we will analyze changes in QoL, mood and burden experimented by principal caregivers and we will attempt to correlate these with changes in different aspects of the disease (motor symptoms, mood, cognition, behavior, other non-motor symptoms, etc.) observed in patients.

A top priority at present in PD is to identify a diagnostic and prognostic biomarker [42, 43]. In COPPADIS-2015, different molecular markers in blood will be evaluated (S-100b protein, TNF-ɑ, IL-1, IL-2, IL-6, vitamin B12, methylmalonic acid, homocysteine, uric acid, C-reactive protein, ferritin and iron) [44–57], and genetic studies (DNA and RNA) [58], and cranial MRI (MTAi, volumetry and tractography) [30, 59–65] will be performed in a subgroup of patients and controls. We will try to identify markers with diagnostic and/or prognostic value, either alone or in combination (clinical and/or paraclinical). For example, we will analyze the role of serum S-100b protein and uric acid as possible prognostic biomarkers [44–46, 56], the relationship between serum levels of TNF-ɑ, IL-1, IL-2, IL-6 and C-reactive protein and different non-motor symptoms [47–51], or the sensitivity and specificity of a simple puzzle test as a cognitive impairment screening test. Moreover, biomarkers included in this project have some of the characteristics needed in an ideal marker: fast and affordable to obtain; available; repeatable; and safe [66, 67]. Finally, in the future, other molecular biomarkers could be analyzed from stored blood samples and other complementary studies could be done. Moreover, creation of a brain bank (COPPADIS Brain Donation Program) from patients included in this project is being developed.

Therefore, well-designed, longitudinal prospective studies must be conducted to identify a biomarker of linear progression and also to understand the natural progression of PD. Today, we still do not know what relationship exists between the progression of motor symptoms and non-motor symptoms in the long term. Different longitudinal studies with prospective follow-up of patients have provided an understanding of the development of motor complications and their relationship with the type of symptomatic therapy initially used [68–70], cognitive impairment and/or dementia [71–74], the course following a given intervention (e.g., deep brain stimulation) [75, 76] or other data on the course of the disease [77–86]. There are other promising studies currently underway with a fundamental objective of identifying a disease progression biomarker [87]. Nevertheless, many of the studies have significant limitations, such as an insufficient sample size and follow-up, significant losses to follow-up, limitations in the recruitment or origin of the sample and performance of analyses not specifically allowed for in the design. As seen for other neurodegenerative diseases like Huntington’s disease [88], the most beneficial study model for achieving relevant advances in the understanding of PD is likely to be a study that includes a baseline cross-sectional assessment of patients (with a control group) with analysis of multiple variables (clinical and complementary tests; for example, serum molecular markers, other biological samples like cerebrospinal fluid, saliva or skin, structural neuroimaging, functional neuroimaging, ophthalmological studies, neurophysiological tests, etc.) followed by a subsequent prospective follow-up (ideally over a long period of follow-up and without losses of cases) of the population studied with multiple periodic analyses that would make it possible to compare the course of the different variables analyzed. These features are all covered by the described project, COPPADIS-2015. Unlike the PPMI Study, PD patients at all *Hoenh & Yahr* stages who meet the selection criteria and not only early patients will be included in the COPPADIS Study. That’s because we focused firstly on studying very exhaustively a large population of PD patients from different areas of Spain analyzing the relationship between different variables according to cross-sectional study methodology. Changes observed in different variables during the follow-up will be adjusted and interestingly, different groups of subjects according to disease duration, motor stage or to have motor complications at baseline could be defined to compare evolution. Some original aspects in COPPADIS are to include the caregivers (the principal caregiver of the patient was also included in a previous Spanish Study, the ELEP Study –*Estudio Longitudinal de pacientes con Enfermedad de Parkinson*/Longitudinal Parkinson’s Disease Patient Study [89] –), to study the incidence and predictors of acute hospitalization and to analyze the value of the MTAi. Furthermore, as it was mentioned, we are working to develop a COPPADIS Donation Brain Program. Indeed, the long-term goal is to design a Spanish PD patients clinical-pathological registry (with the inclusion of early new patients) with long-term monitoring and implementation of many additional tests.

In conclusion, COPPADIS-2015 is a challenging and original initiative. We hope that this project will provide important information regarding the natural history of PD, including changes in motor and non-motor symptoms, QoL and caregiver burden over time, and the value of various biomarkers.

## Abbreviations

ADLS: Schwab & England activities of daily living scale
AEMPS: Agencia Española del Medicamento y Productos Sanitarios
BDI: beck depression inventory
CSI: caregiver strain index
e-CRF: electronic case report form
EUROHIS-QOL: European health interview survey-quality of life
FOGQ: freezing of gait questionnaire
HY: hoenh & yahr
IL: interleukin
MMSE: mini mental state examination
MRI: magnetic resonance imaging
MTAi: medial temporal atrophy index
NMSS: non-motor symptoms scale
NPI: neuropsychiatric inventory
PD: parkinson’s disease
PD-CRS: parkinson’s disease cognitive rating scale
PDQ-39SI: 39-item Parkinson’s disease quality of life questionnaire summary index
PDSS: parkinson’s disease sleep scale
QoL: quality of life
QUIP-RS: questionnaire for Impulsive-compulsive disorders in parkinson’s disease-rating scale
TNF: tumor necrosis factor
UPDRS: unified parkinson’s disease rating scale
VAFS: visual analog fatigue scale
VAS-Pain: visual analog scale-pain
ZCBI: zarit caregiver burden inventory
