# Erratum to: COPPADIS-2015 (COhort of Patients with PArkinson’s DIsease in Spain, 2015), a global –clinical evaluations, serum biomarkers, genetic studies and neuroimaging– prospective, multicenter, non-interventional, long-term study on Parkinson’s disease progression

**DOI:** 10.1186/s12883-016-0567-6

**Published:** 2016-04-06

**Authors:** Diego Santos-García, Pablo Mir, Esther Cubo, Lydia Vela, Mari Cruz Rodríguez-Oroz, Maria José Martí, José Matías Arbelo, Jon Infante, Jaime Kulisevsky, Pablo Martínez-Martín, COPPADIS Study Group

**Affiliations:** Sección de Neurología, Complejo Hospitalario Universitario de Ferrol (CHUF), Hospital Arquitecto Marcide, c/Avenida La Residencia, s/n, Ferrol, 15405 Spain; Unidad de Trastornos del Movimiento, Servicio de Neurología y Neurofisiología Clínica, Instituto de Biomedicina de Sevilla, Hospital Universitario Virgen del Rocío, CSIC y Universidad de Sevilla, Sevilla, Spain; Centro de Investigación Biomédica en Red sobre Enfermedades Neurodegenerativas (CIBERNED), Sevilla, Spain; Servicio de Neurología, Hospital Universitario de Burgos, Burgos, Spain; Unidad de Neurología, Fundación Hospital de Alcorcón, Madrid, Spain; Instituto de Investigación Biodonostia, Hospital Universitario Donostia, San Sebastián, Spain; Unidad de Parkinson y Trastornos del Movimiento, Servicio de Neurología, Instituto Clínico de Neurociencias, Hospital Clínic, Barcelona, Spain; Unidad de Trastornos del Movimiento y enfermedad de Parkinson, Servicio de Neurología, Hospital Universitario Insular de Gran Canaria, Las Palmas de Gran Canaria, Spain; Unidad de Trastornos del Movimiento, Servicio de Neurología, Hospital Universitario Marqués de Valdecilla, Santander, Spain; Unidad de Trastornos del Movimiento, Servicio de Neurología, Hospital de Sant Pau, Barcelona, Spain; Centro Nacional de Epidemiología, Instituto de Salud Carlos III, Madrid, Spain

## Erratum

After publication of the original article [1], it has come to the authors’ attention that there were a number of errors in the manuscript which were not identified at proofing. These errors affect the affiliations, Table 2, and the COPPADIS study group collaborators listed in the Acknowledgements section.

**Table 2:** Centers, number of patients that they have estimated to recruit and distribution of patients selected for the complementary studies according to the different centers

Principal Investigator; PI’s participating site		City	Patients (n)	Controls (n)	Complementary studies (PDP/CS)
1.	Diego Santos García; Neurology Section, Hospital Arquitecto Marcide, Complejo Hospitalario Universitario de Ferrol (CHUF).	Ferrol (A Coruña)	60	10	45/10
2.	(1) Oriol de Fábregues-Boixar Nebot and (2) Jorge Hernández Vara; Movement Disorders Unit, Neurology Service, Hospital Universitario Vall d’Hebron.	Barcelona	60 (40/20)	20 (10/10)	40/20
3.	Carmen Borrue Fernández; Movement Disorders Unit, Neurology Service, Hospital Infanta Sofía.	Madrid	50	10	NO
4.	Pablo Mir Rivera; Movement Disorders Unit, Neurology and Clinical Neurophysiology Service, Instituto de Biomedicina de Sevilla, Hospital Universitario Virgen del Rocío, CSIC and Universidad de Sevilla.	Seville	40	10	40/0
5.	Maria José Martí Domenech; Parkinson’s and Movement Disorders Unit, Neurology Service, Instituto Clínico de Neurociencias, Hospital Clínic.	Barcelona	40	10	NO
6.	Miquel Aguilar Barberá; Movement Disorders Unit, Hospital Universitario Mutua de Terrassa.	Barcelona	40	10	30/10
7.	Beatriz Tijero Merino; Functional Neurology and Parkinson’s Disease Unit, Hospital de Cruces.	Bilbao	35–40	10	NO
8.	José Chacón Peña; Neurology Unit, Hospital Infanta Luisa.	Seville	30–35	10	NO
9.	(1) Manuel Seijo Martínez and (2) Iria Cabo López; Neurology Section, Hospital de Pontevedra.	Pontevedra	30 (15/15)	20 (10/10)	NO
10.	Víctor Puente Périz; Movement Disorders Unit, Neurology Service, Hospital del Mar.	Barcelona	25–30	10	20/10
11.	Inés Legarda Ramiréz; Neurology Service, Hospital Universitario Son Espases.	Palma de Mallorca	25	10	NO
12.	Francisco Carrillo Padilla; Neurology Service, Hospital Universitario de Canarias, San Cristóbal de la Laguna.	Santa Cruz de Tenerife	25	10	20/0
13.	Lydia López Manzanares; Movement Disorders Unit, Neurology Service, Hospital La Princesa.	Madrid	25	10	NO
14.	Caridad Valero Merino; Neurology Unit, Hospital Arnau de Vilanova.	Valencia	20–25	10	NO
15.	Jaime Kulisevsky Bojarski; Movement Disorders Unit, Neurology Service, Hospital de Sant Pau.	Barcelona	20	10	NO
16.	José Manuel García Moreno; Movement Disorders Unit, Hospital Universitario Virgen Macarena.	Seville	20	10	20/10
17.	Benito Galeano Bilbao; Neurology Section, Hospital Universitario de Ceuta.	Ceuta	20–25	10	20/0
18.	Nuria Caballol Pons; Movement Disorders Unit, Consorci Sanitari Integral, Hospital Moisés Broggi.	Sant Joan Despí	20	10	NO
19.	Mari Cruz Rodríguez Oroz; Hospital Universitario Donostia, Instituto de Investigación Biodonostia.	San Sebastián	20	10	20/10
20.	María Iciar Gastón Zubimendi; Movement Disorders Unit, Neurology Service, Complejo Hospitalario de Navarra.	Pamplona	20	10	NO
21.	Pilar Sánchez Alonso; Neurology Service, Hospital Puerta de Hierro.	Madrid	15–25	10	NO
22.	Esther Cubo Delgado; Neurology Service, Complejo Asistencial Universitario de Burgos.	Burgos	15	10	NO
23.	Lydia Vela Desojo; Neurology Unit, Fundación Hospital de Alcorcón.	Alcorcón (Madrid)	15	10	NO
24.	Maria José Catalán Alonso; Movement Disorders Unit, Neurology Service, Hospital Clínico San Carlos.	Madrid	15	10	NO
25.	Luis Manuel López Díaz; Neurology Section, Hospital da Costa.	Burela (Lugo)	15	10	NO
26.	Maria Gema Alonso Losada; Neurology Service, Hospital Meixoeiro, Complejo Hospitalario Universitario de Vigo (CHUVI).	Vigo (Pontevedra)	15	10	NO
27.	Nuria López Ariztegui; Movement Disorders Unit, Complejo Hospitalario de Toledo.	Toledo	15	10	NO
28.	Mónica Kurtis Urra; Movement Disorders Unit, Neurology Service, Hospital Ruber Internacional.	Madrid	15	10	NO
29.	Jon Infante Ceberio; Movement Disorders Unit, Neurology Service, Hospital Universitario Marqués de Valdecilla.	Santander	15	10	15/10
30.	Sonia Escalante Arroyo; Neurology Service, Hospital de Tortosa Verge de la Cinta (HTVC).	Tortosa (Tarragona)	15	10	15/10
31.	Juan Carlos Martínez Castrillo; Movement Disorders Unit, Neurology Service, Hospital Ramón y Cajal.	Madrid	15	10	NO
32.	José Matías Arbelo González; Movement Disorders and Parkinson’s Disease Unit, Neurology Service, Hospital Universitario Insular de Gran Canaria.	Las Palmas de Gran Canaria	15	10	NO
33.	René Ribacoba Montero; Movement Disorders Unit, Neurology Service, Hospital Central de Asturias.	Oviedo	15	10	15/10
34.	Jessica González Ardura; Neurology Service, Hospital Universitario Lucus Augusti (HULA).	Lugo	15	10	NO
35.	Javier López del Val; Movement Disorders Unit, Neurology Service, Hospital Clínico Universitario Lozano Blesa.	Zaragoza	15	10	NO
36.	María Asunción Ávila Rivera; Movement Disorders Unit, Consorci Sanitari Integral, Hospital General de L’Hospitalet.	L’Hospitalet de Llobregat	15	10	NO
37.	Hortensia Alonso Navarro; Neurology Section, Hospital Universitario del Sureste, Madrid.	Madrid	15	10	NO
38.	Berta Solano Vila; Neurology Service, Hospital Josep Trueta and Parc Martí i Juliá, Girona.	Girona	15	10	15/10
39.	Juan García Caldentey; Neurology Unit, Centro Neurológico OMS 42.	Palma de Mallorca	15	10	NO
40.	Ana Rojo Sebastián; Parkinson’s and Abnormal Movement Unit, Neurology Service, Hospital Universitario Príncipe de Asturias.	Alcalá de Henares (Madrid)	15	10	NO
41.	Silvia Martí Martínez; Neurology Service, Hospital General de Alicante.	Alicante	15	10	NO
42.	José Andrés Domínguez Morán; Neurology Unit, Hospital de la Rivera.	Alcira (Valencia)	15	10	NO
43.	Irene Martínez Torres; Movement Disorders Unit, Neurology Service, Hospital La Fe.	Valencia	15	10	NO
44.	María Álvarez Sauco; Neurology Service, Hospital General Universitario de Elche.	Elche (Alicante)	15	10	NO
45.	Cristina Prieto Jurczynska; Movement Disorders Unit, Hospital Infanta Elena-Hospital Rey Juan Carlos-Hospital Collado Villalba, Madrid.	Madrid	15	10	NO
			1.000	470	315/110

In the author list, J. Infante should have been affiliated with institutions 3 and 9, rather than just 9, as appears correctly in the author list of this erratum.

There were a number of errors in Table 2, involving the names of institutions listed in column 1. Minor errors were present in institutions 25 (Hospital da Costa, Burela), and 39 (Centro Neurológico OMS 42) within the table. In addition, the ‘Complementary studies’ column of institution 38 (Hospital Josep Trueta and Parc Martí i Juliá) should have read ‘15/10’. As a result, the row of totals found at the bottom of Table 2 was incorrect for this column, and should have read ‘315/110’.

Table 2 appears correctly in this erratum.

Finally, there were errors in the COPPADIS Study group (collaborators) section. The sentence “Finally, this author will not participate in the study” was mistakenly included between the names of Ferriol MM and Leiva C. In addition, the collaborator Clemente J should have appeared as ‘Segundo JC’.

Within the affiliations of this section, the city names of institutions 19 (Hospital Clínico San Carlos, Madrid) and 20 (Hospital da Costa, Burela, Lugo) appear incorrectly.
